# Loneliness and coping strategies in older adults in nursing homes in the Northern region of Malaysia: A mixed-method study

**DOI:** 10.1371/journal.pone.0349148

**Published:** 2026-06-03

**Authors:** Ahmad Zambri Ilham, Mohd Noor Norhayati, Ismail Shaiful Bahari

**Affiliations:** Department of Family Medicine, School of Medical Sciences, Universiti Sains Malaysia, Kubang Kerian, Kelantan, Malaysia; No institution, UNITED KINGDOM OF GREAT BRITAIN AND NORTHERN IRELAND

## Abstract

**Background:**

Loneliness among institutionalized older adults poses significant health risks, yet remains understudied. A deeper understanding can help shape more compassionate, culturally appropriate, and effective care approaches for the aging demographic. This study aimed to explore the lived experiences of loneliness and the coping mechanisms adopted by older adults residing in institutionalized nursing homes in the Northern region of Malaysia.

**Methods:**

A mixed-method study was used. A qualitative phenomenological approach was conducted at Rumah Seri Kenangan Taiping, Perak, Malaysia. Participants were selected via purposive sampling. Data collection comprised face-to-face semi-structured interviews. A quantitative approach using the Malay version of the 6-item De Jong Gierveld Loneliness Scale triangulates the methods. Thematic analysis was applied to the interview transcripts using NVivo version 14, and descriptive statistics were performed in SPSS version 27 to analyze loneliness scale scores.

**Results:**

Twenty-three older adults participated in both qualitative and quantitative analyses. All participants experienced loneliness; 13 respondents were most lonely, with a mean (SD) loneliness score of 4.52 (1.04). Five causes contributing to loneliness were identified: loss of loved ones, health-related issues, socio-economic challenges, socio-cultural changes, and environmental restrictions within the institution. Loneliness impacted participants’ physical health, emotional well-being, and mental health. Coping strategies clustered into three themes: spiritual coping (religious fulfillment and acceptance), strengthening social ties (contact with family, bonding with residents, and visitor interactions), and attention distraction (hobbies, social media use, and participation in institutional activities).

**Conclusions:**

Loneliness among institutionalized older adults is a multidimensional experience with significant physical and emotional repercussions. However, participants demonstrated resilience through spiritual, social, and behavioral coping strategies. These findings underscore the importance of holistic interventions that enhance emotional support, spiritual well-being, and meaningful engagement to improve the quality of life for older adults living in institutional settings.

## Introduction

Life expectancy at birth is expected to rise from 72.8 years in 2019 to 77.2 years in 2050 [1]. On a global scale, the population aged 65 and above is experiencing a more rapid growth compared to other age groups. Furthermore, projections indicate a threefold increase in the population aged 80 and over, from 143 million in 2019 to an estimated 426 million by the year 2050 [2]. The estimated total population of Malaysia in 2023 reached 33.4 million, marking a rise from 32.7 million in 2022, with an annual population growth rate of 2.1%. Malaysia is facing an imminent challenge of an aging population, which is projected to materialize as early as 2030 [3].

Loneliness is subjective distress resulting from a discrepancy between desired and actual relationships [4]. It is a negative and subjective experience resulting from the cognitive evaluation of a perceived mismatch between the quantity and quality of existing relationships and one’s relational standards [5]. Older adults who are more than 60 years old [2,6] are prone to loneliness due to worsening physical health, loss of family and friends, and social isolation [7].

Loneliness is a common concern among older Malaysians, with more than half of older adults (53.4%) experiencing loneliness [8]. In a cross-sectional study involving 80 older adults from nursing homes in Pahang, Malaysia, it was observed that all participants experienced loneliness. Among them, 25% reported moderate loneliness, while the remaining 75% reported profound loneliness [9]. Loneliness and social isolation were shown in a meta-analysis to be associated with an increased risk of mortality of 26% and 29%, respectively [10]. The presence of loneliness increased the risk of heart disease by 9%, the risk of stroke by 32%, and the risk of dementia by 50%. The long-term effects can be more severe, leading to elevated blood pressure, weight gain, increased engagement in smoking, alcohol/drug use, and diminished cognitive function [11].

Loneliness and social isolation are significant problems that have an adverse effect on the physical and mental health and longevity of older adults [12]. Social loneliness arises when an individual lacks a sense of social integration or community engagement, resulting in a breakdown of social connectedness [13]. Loneliness and symptoms of depression can synergistically contribute to a decline in well-being among middle-aged and older adults [14]. Despite experiencing developmental changes such as losses of social relationships, social roles, and functional ability, older adults can often remain resilient by employing specific coping strategies [15].

Coping is defined as people’s cognitive and behavioral efforts to manage specific demands appraised as taxing or exceeding their resources [16]. The coping mechanisms for older adults differ from those of other age groups, given that stressors evolve with age [17]. The interplay of macro-societal elements like social resources, economic circumstances, cultural norms, technological accessibility, urban planning, and other worldwide phenomena like migration in determining people's feelings of connection or isolation is encapsulated in the infrastructure of loneliness. Understanding this infrastructure is essential to developing strategies to alleviate loneliness among older adults [18].

Nearly half of Malaysian older adults face the risk of social isolation, causing significant implications for their physical, mental, and emotional well-being. Despite the increasing recognition of loneliness as a public health issue, there remains a gap in understanding the root causes, impact, and effective interventions to address this pervasive issue among the older adult population. Addressing loneliness is crucial not only for their emotional well-being but also for the prevention and management of a range of serious health conditions, including cardiovascular diseases, dementia, depression, anxiety, and other mental health issues [19]. The current study aimed to explore the lived experience and uncover the strategies for combating loneliness among institutionalized older adults in the Northern region of Malaysia. The findings help us better understand the root cause and improve the approach to tackling loneliness among older adults in Malaysia.

## Materials and methods

### Population and sample

A qualitative research study, specifically a phenomenological approach, is considered appropriate, as it explores people’s life experiences [20]. Rumah Seri Kenangan Taiping, a government-run institution for older adults managed by the Social Welfare Department in Taiping, Perak, Malaysia, was chosen as the study area. This shelter home caters to older adults in Kedah, Pulau Pinang, and Perak, which are located in the Northern region of Malaysia. Older adults aged 60 years or older were included. Those who were unable to communicate in Bahasa Malaysia and diagnosed with any psychiatric illness, dementia, or any other severely disabling illnesses were excluded.

The research used purposive sampling, intentionally selecting respondents who could provide pertinent information. Participation was voluntary for those who fulfilled the inclusion and exclusion criteria. The Principal Investigator (AZI) identified participants based on their known diagnoses, such as hypertension, diabetes mellitus, and dyslipidemia, with assistance from nursing home staff; however, no specific diagnoses were included in the inclusion criteria. This applies only to the exclusion criteria. The number of older adults included in this study was based on saturation theory, whereby the number of interviews increases until no new themes emerge from the respondents or until no new information can be obtained [21]. In phenomenological studies, a sample between five and 25 is considered adequate, and 15 is the smallest acceptable sample [22].

### Research tools

This study utilized a semi-structured interview guide to elicit information by asking prepared questions while at the same time allowing respondents to elaborate on their responses [23]. It is dominated by open-ended questions rather than closed-ended ones to allow for unexpected turns that follow the respondents’ interest or knowledge. Respondents were asked about factors causing their loneliness, their impacts on them, and how they overcame their feelings of loneliness. Further questions were asked to elaborate more based on each of their responses.

To reduce subjectivity, a quantitative approach using the 6-item Malay version of the De Jong Gierveld Loneliness Scale is utilized to triangulate the methods [24]. The questionnaires were administered to the same participants at the beginning of the interviews, which lasted less than 5 minutes. The scale consisted of two subscales, i.e., emotional (3 items) and social (3 items). It encompassed three negatively formulated items (‘I experience a general sense of emptiness’, ‘I miss having people around’, and ‘Often, I feel rejected’) and three positively formulated items (‘There are plenty of people that I can lean on in case of trouble’, ‘There are many people that I can count on completely’ and ‘There are enough people that I feel close to’). Respondents rated their responses to statements on a 5‐point Likert scale with options including ‘yes!’ ‘yes,’ ‘more or less,’ ‘no’ and ‘no!’ A total score was calculated after subscale responses were recorded to account for negatively worded items, providing a range of zero (not lonely) to six (very lonely) [12,24]. It has adequate psychometric properties such as good internal consistency, test‐retest reliability, and convergent validity. The Cronbach’s alpha internal consistency coefficient of Bahasa Malaysia’s De Jong Gierveld Loneliness Scale was 0.71. A value of more than 0.7 indicates a measure of acceptable internal consistency [25].

### Data collection

Data were collected from October 20, 2024, to January 20, 2025. The screening for inclusion and exclusion criteria, the explanation of the study purpose and procedures, and the obtaining of verbal and written consent were performed (AZI).Interviews were done one-to-one in a nursing room where privacy was maintained and external interruptions were minimized throughout the interviews. Face-to-face in-depth interviews were conducted. We used a semi-structured interview guide followed by the Malay version of the De Jong Gierveld Loneliness Scale. Each interview lasted for approximately 45 minutes to one hour. The entire interviews were audiotaped and transcribed verbatim in the respondents’ language. The audio recording was anonymous, and no personally identifiable information was mentioned during the interview. Any missing information was checked and updated. Any unclear information was clarified with the respondents. Written informed consent was signed prior to the interview.

### Data analysis

A descriptive analysis of the De Jong Gierveld Loneliness Scale was performed using SPSS version 27. The verbatim transcripts were managed using computer-aided qualitative data analysis (CAQDA) software, NVIVO version 14 (QSR International Pty Ltd., 2012), using thematic data analysis [26]. This research uses thematic analysis and an inductive type of coding process. Researchers read the data, identify patterns, and create nodes as themes naturally emerge. The analysis took place concurrently with the data collection and continued until the saturation point was reached. The participants were identified by an identification number to maintain the confidentiality of the findings. Data analysis was done by the principal investigator and co-researcher. The coding for the same text is cross-checked and agreed to by the co-researcher. Reflexivity was maintained by regular debriefing sessions with peers throughout the research, enhancing the credibility and trustworthiness of the qualitative study.

Rigor is attained by implementing reliability and validity in the qualitative inquiry [27]. Reliability was achieved in this study by reading and re-reading the transcript to familiarize the researchers and ensure their overall meanings were understood. The transcripts were checked by listening to the audio recordings to ensure errors were not introduced during transcription and coding. During the coding process, data were constantly compared with codes to ensure no changes in the meaning of the codes occurred. The coding for the same text is cross-checked and agreed to by a co-researcher.

Validation is attained in this study by applying triangulation to enhance the research’s credibility. The interview was supported by the Malay version of the De Jong Gierveld Loneliness Scale questionnaire to strengthen the accuracy of information by reducing subjectivity and to make the qualitative phases of research more objective and credible. Through the member-checking process, the polished content of the transcripts and the quotations cited were read to the respondents. This is done by rechecking the interviews after they were transcribed and the themes were constructed. Rich, thick description was used to provide many perspectives about a theme so that the results become realistic and richer.

### Ethics approval

This study has received ethical approval from the Human Research Ethics Committee of Universiti Sains Malaysia (USM/JEPeM/24050390) and adheres to the Helsinki Declaration principles. Several measures were taken to address their vulnerability, which include the following: (i) the respondents were informed that their participation in the research was voluntary. It is permissible for them to withdraw at any time. (ii) The respondents were free to withdraw from this study at their discretion. The reason for termination may be asked whether it relates to the research. (iii) The consent forms emphasized the risk, confidentiality, privacy, and termination method. The audio recording was for transcription purposes and will not be copied or sent to any other individual or used for any other purpose. (iv) The respondents were informed that the researchers would terminate this research if there were any safety issues.

## Results

A total of 25 participants were selected by nursing home staff, and all consented to the research. Two participants were excluded as they met the exclusion criteria. No dropouts occurred. Data saturation was reached at participant 20, with three additional interviews conducted to ensure robustness. Therefore, 23 respondents with a mean age of 70 years were interviewed. Table 1 shows the sociodemographic characteristics of older adults in the nursing home.

**Table 1:** Sociodemographic characteristics of older adults in the nursing home (n = 23).

Variable	n	
Age (year)	70.0	(7.41)^a^
Sex		
Male	17	
Female	6	
Marital status		
Single	10	
Divorced	6	
Widowed	7	
Comorbidities		
Present	17	
Absent	6	
Educational level		
No formal education	5	
Primary	7	
Secondary	11	
Income per month		
No income	10	
<RM 1000	7	
RM 1000 – RM 3000	5	
> RM 3000	1	
Activities of daily living		
Independent	17	
Dependent	6	

^a^ Expressed as mean (standard deviation)

Loneliness based on the Malay version of the De Jong Loneliness Scale item 1 (*experience a general sense of emptiness*) reported the highest reason for loneliness with a mean (SD) of 0.96 (0.21), followed by item 5 (*a lack of people that they can trust*) with the mean (SD) of 0.87 (0.34). Most participants (56.5%) fall into the ‘most lonely’ category (score 5–6), while 43.5% are classified as ‘moderately lonely’ (score 3–4). None of the participants fell into the ‘least lonely’ category. The mean (SD) of the total loneliness score was 4.52 (1.04) (Table 2).

**Table 2:** Description of loneliness based on the Malay version of the De Jong Gierveld Loneliness Scale (n = 23).

Item	Variables	Mean	(SD)^a^
1	I experience a general sense of emptiness.	0.96	(0.21)
2	I miss having people around.	0.78	(0.42)
3	I often feel rejected.	0.57	(0.51)
4	There are less people I can rely on when I have problems.	0.74	(0.45)
5	There are less people that I can trust completely.	0.87	(0.34)
6	There are less people that I feel close to.	0.61	(0.50)

^a^ Standard deviation

The wordings for the positively formulated items (4–6) were reversed for clearer understanding.

The thematic analysis in Table 3 identifies key factors contributing to loneliness, its impacts, and coping strategies. Themes under factors of loneliness include loss of loved ones, health-related, socio-economic, socio-cultural, and environmental factors. Physical, mental, and emotional health are themes that impact loneliness. Themes for coping strategies include spiritual coping, strengthening social ties, and attention distraction.

**Table 3:** Themes and subthemes for older adults with loneliness.

Themes	Subthemes
***Causes of loneliness***	
Loss of loved ones	(i) Death of children/parents/siblings
	(ii) Spouse passed away
Health-related	(i) Chronic diseases
	(ⅱ) Physical limitations
Socio-economic	(i) Financial issues
	(ⅱ) Job termination
Socio-cultural	(i) Commitment of other family members
	(ii) Living as a vagabond
	(ⅲ) Loss of contact with friends/family members
Environmental factors	(i) Having no visitors
	(ii) Limited access to sources or activities
	(ⅲ) Life in confinement
	(ⅳ) Limited circle of friends
***Impacts of loneliness***	
Physical health	(i) Feeling tired
	(ii) Lack of appetite
	(ⅲ) Sleep disturbance
Mental and emotional	(i) Feeling loss and sad
	(ii) Lack of motivation
	(ⅲ) Shock of death
	(ⅳ) Feeling down and lonely
	(v) Feeling of worry
***Coping strategies***	
Spiritual coping	(i) Religious fulfilment
	(ii) Contentment
Strengthening social ties	(i) Admission to welfare home
	(ii) Contact with families and friends
	(ⅲ) Visitors at welfare home
	(ⅳ) Relationship with other occupants
Attention distraction	(i) Engagement in social media and hobbies
	(ii) Activities with the welfare homes
	(ⅲ) Professional aid and support

### Causes of loneliness

The first theme describes the causes of loneliness. It is the judgment of the participants on factors contributing to their loneliness. Findings related to the causes of loneliness fell into four subthemes: loss of loved ones, health-related, socio-economic, socio-cultural, and environmental factors. ***Loss of loved ones:*** Most participants claimed that the loss of loved ones contributed to their loneliness. One participant mentioned living alone after his only sister passed away (A004). The other seven participants shared that almost all their siblings had passed away. Many of the participants missed their parents and became lonely after their passing. Two of them felt lonely after they lost their mother.

*“I used to have my mother… but she was no longer around. They sent me to stay in a nursing home.”* (A010)

Two participants felt lonely after losing their children. One of them kept thinking of her foster son, who passed away (A015). Six participants talked about how they became lonely after their spouse passed away. They were close to each other, and the loss of their spouse significantly contributed to their loneliness. One participant said that after his wife passed away, he started to feel very lonely.

*“After that, she passed away… she passed away… and after she died, that is when I started feeling very lonely.”* (A002)

***Health-related:*** Having chronic diseases and physical limitations were some of the factors that contributed to loneliness. Most participants involved have underlying chronic illnesses, namely hypertension, stroke, diabetes, enlarged prostate, high cholesterol, heart disease, and asthma, and these illnesses somehow contributed to their loneliness. One participant claimed that his health problems, including a stroke, made him lonely (A006). Another participant said that his diabetes was the one causing all of the issues, leading to his loneliness.

*“It is because… I have diabetes. I was admitted to the hospital, and my next of kin… we have lost contact for some time. But for me… I feel that most of my problems now are mainly due to this diabetes… it has affected me a lot.”* (A003)

More than half of the participants said that their physical limitations are one factor in their loneliness. Physical limitations restrict their movements and freedom to do things they used to like. One patient said his stroke makes him unable to walk; he needs help with everything, and he feels like he cannot do anything.

*“My legs cannot walk. I need help with everything. I cannot even walk… I cannot do anything at all.”* (A004)

The other participant claimed she could not cook anymore, which she used to love, since she had the stroke (A016). One participant claimed he felt lonely after an accident. He could not work anymore and quit his job. Since the accident, he felt lonely and filled with emptiness (A023). ***Socio-economic:*** Before admission to the nursing home, their socio-economic status plays a significant role in their life. Socio-economic factors contributing to their loneliness include having financial issues and job termination. With the current higher standard of living, many participants find it challenging to earn a decent salary. A few did multiple odd jobs, and some got their employment terminated. Almost half of the participants said that financial issues were one of the factors; one participant said that everything needed to be paid for nowadays to support the family and house maintenance.

*“If there is not enough money, I cannot support the family… food and everything needs to be paid for. Gotta pay for electricity, water… have to pay for everything.”* (A001)

***Socio-cultural:*** Commitment of other family members, living as a vagabond, and loss of contact with friends and family members are a few factors that contributed to their loneliness. Almost half of the participants were never married and lived with family members or siblings with their families and commitments. One participant said that after his wife passed away, he stayed alone and did not want to burden his children or family members.

*“Feeling lonely... It’s because of the passing, right… Umm... other family members also seem to have their own commitments... the children have to take care of their own kids. Everyone has their own families, and I don’t want to trouble anyone. Even working... I’m no longer strong enough to earn my own income.”* (A002)

Some of the participants lived on the streets as vagabonds because they did not have anyone to take care of them until they were brought to the nursing home by the authorities.

*“I left my inherited house… I used to wander around for a year and a half, staying on sidewalks… I slept on the sidewalks… ate rice… I sold boxes… there was some help from the community.”* (A020)

Some participants claimed they were lonely because they had no children or had lost contact with their children or family members.

*“Sometimes at night… I think about my fate… not having any children… ending up like this… sometimes I talk to the nurses… I feel lonely.”* (A009)

***Environmental factors:*** Having no visitors, limited access to sources or activities, having a restricted circle of friends, and life in confinement were some of the factors that were highlighted. Almost half of the participants never had visitors visiting them at the nursing home. Few participants shared that living in a nursing home has limited access to resources or activities. There was not much to be done, and they preferred to lie down and rest on their bed. Half of the participants said that living in the nursing home was like living in confinement, making them feel bored and lonely.

*“I kept thinking... I used to love being free... staying here makes me feel restricted... suffocated. Sometimes, back when I had a motorcycle... I would ride around Baling, Sik, Gerik... I loved seeing nature... the forests. Now it feels confined... like being in a prison. If I did not have a stroke, I would not have come to this old folks’ home... I would have lived on my own. It is lonely, restricted, feels forced, and not free.”* (A016)

The circle of friend was also limited, given their small community in the nursing home. Most of them come from different families and socio-economic backgrounds, making it harder to find a similar topic of interest, and they preferred to keep it to themselves.

*“I do not really talk to people here... I stay quiet. When we talk… There is no idea. No topic. It’s different when working… we have many work-related topics. But here… I do not know them… I do not know many of them.” (A023)*

### Impacts of loneliness

This theme describes the impacts of loneliness on the participants, which has two subthemes: physical health and mental and emotional well-being. ***Physical health:*** This includes feeling tired, a lack of appetite, and having sleep disturbances. One of the participants has been feeling exhausted most of the time, mainly because he lies down for too long, making him even more tired and eventually making him feel sick (A005). A few participants lacked appetite; some attributed this to old age, while others did not feel like eating as they kept thinking of their loved ones.

*“So I feel troubled... I cannot... when I see other people’s children coming to visit... I become... yeah... sad. No appetite to eat... just looking... when I see other people’s children coming back, I feel sad.”* (A015)

More than half of the participants experienced sleep disturbances, as they kept thinking of their old life and the uncertainties of their future life, especially at bedtime.

*“Sleep is not very restful... sometimes I cannot sleep well either... I keep thinking... waking up around 3... in the morning... just thinking... if I were to die tomorrow, where would it happen?”* (A014)

***Mental and emotional:*** The participants described the impacts of loneliness on their mental and emotional well-being, including feeling lost and sad, lack of motivation, the shock of death, feeling down and lonely, and feeling worried. A few of the participants describe how the experience made them think of loss and emptiness. One participant felt loss after their spouse passed away.

*“Yeah... after she passed away... I felt a real sense of loss, you know... because we went through all the ups and downs together back then.” (A020)*

More than half of the participants said they have been feeling sad, mostly when thinking about their old life, losing their loved ones, financial issues, and uncertainties in life. One participant had been feeling sad thinking of how her old life used to be and how life was easy back then (A013). Eight participants said that they lack the motivation to do anything inside the nursing homes, that there are limited activities to be done, and that, in the end, they do not feel like doing anything anymore. One participant said that after a while, he became frustrated and fed up with everything and preferred doing nothing (A003). A few participants shared that they had been feeling shocked after the death of their loved ones. They were so used to living with their spouse, children, and family members that the sudden loss had a significant impact on them. One participant just collapsed and was shocked after her son passed away in a road traffic accident.

*“When he passed away, the police came in the middle of the night... I did not believe the police... I said... he had just come back. I could not even imagine it. Yeah. After that, they brought me to the scene. They showed me that black bag, I opened it... I could not speak [crying]...I just collapsed... slumped by the roadside.” (*A015)

More than half of the participants shared that they sometimes thought of their old life and how good and happy it was. They miss their old life when they were young and free. These sometimes made them feel down and lonely. One participant said he felt lonely, especially alone (A005). Some participants shared that they could not help but get bored easily staying at the nursing home, as there were not many activities to do, and they were done with playing games, watching television, and listening to the radio. Most preferred to lie in bed, procrastinate, and sleep. Four participants were worried about their health and their uncertain future. One of them was concerned about how he might fall ill and become bedridden, causing a burden to those around him.

*“I am worried... I am afraid that if I fall sick, I will become a burden to others. If you are sick, you must stay in the ward at the back for bedridden patients. In my heart, it is not that I am asking for much... I pray, oh Allah, my Lord... if You are going to take my life... please do not let me suffer through illness.”* (A002)

### Coping strategies

This theme describes the participants’ coping strategies in dealing with their loneliness. Findings related to coping strategies fell into three subthemes: spiritual coping, strengthening social ties, and attention distraction. ***Spiritual coping***: They frequently used religious reasoning, such as reliance and contentment, to manage or adapt to difficult life events, especially when thinking about their old life and the loss of their loved ones. One participant tried to be closer to God by performing prayers and reciting du’a and zikr (remembrance of God) whenever he started to feel lonely and emotional, especially when he was alone.

*“When I am alone, I do not want to give in to emotional stress... so I go... I hold my prayer beads... recite du’a, perform my prayers... When we follow our emotions, we become stressed, bring ourselves closer to Allah, recite a lot of zikr, and feel at peace,... and that creates the atmosphere.”* (A020)

Few participants benefited from attending religious talks and classes held at the nursing home. One participant, in particular, likes to watch religious talks and videos online. Most participants cope by feeling gratitude and contentment with their current life, support system, and belief in God.

*“We stayed in this facility... so be grateful. Here, I get food... I have protection... I can perform my worship... just thinking about that is enough. It is God who has the power. I accept it with a willing heart... we remember Him, the One.”* (A009)

***Strengthening social ties***: Another coping strategy is to improve their social relationship. More than half of the participants said that being admitted to the nursing home and having the care and facilities does help them to cope with their life and loneliness. One said he did not need to worry about money or food anymore. Everything is taken care of by the nursing home.

*“No need to worry anymore… you can eat everything. Yeah… it is even better to stay here. Yeah… no more being lonely. After entering the welfare home, things got better.”* (A001)

Most participants felt happy and less lonely whenever their relatives or friends came and visited them at the nursing home. A few still keep in touch with their family members via phone calls and text messages. One participant was happy whenever he had visitors because he could catch up and talk about how things had changed over the past years, and how they would give him pocket money and bring him some things (A020). Another participant felt less lonely whenever her sibling came and visited her. She sometimes would follow and have a staycation at their house.

*“They come quite often… if I call, they will come… sometimes they invite me to their house… I go along… sometimes for a week. Yeah… it is okay… I feel less lonely.”* (A011)

Having good relationships with other occupants helped most participants cope with daily life. Sharing their old life stories and talking about life improved their mood and motivation. One participant said he had a lot of help and assistance from fellow occupants when he first had a stroke.

*“Yeah… back then, when I first had a stroke, my friends here helped. They helped push me slowly… whatever needed to be done, they helped.”* (A004)

Another participant said he and the other occupants had become a family compared to being alone outside before coming to the nursing home (A008). ***Attention distraction:*** participants describe another coping strategy to combat loneliness as distracting their attention, by engaging in social media and hobbies, doing activities with the nursing home, and professional aid and support. Four participants said engagement in various social media platforms helped them see the outside world, interact with online friends, and put their minds at ease.

*“I have TikTok… all sorts of things come up… I watch YouTube too. I also have Facebook. I do have acquaintances… many, but not… not close ones.”* (A005)

Most participants distracted their attention by doing hobbies and activities that they love, such as reading books, magazines, and newspapers. Apart from that, they enjoyed watching television and listening to the radio. Some played with their mobile phones, surfed the internet, or played mobile games. Few participants love playing games at the nursing home, such as darts, carrom, and ping pong. Some participants preferred doing light exercises, helping with cleaning the areas, doing some simple gardening, and helping with kitchen chores.

*“I do a bit of work… any kind of work… like tidying up the area… to get rid of the loneliness… also do some exercises and all, you know. No pain at all… feeling good.”* (A008)

A few other participants preferred talking to one another or just going to sleep whenever they felt bored. Five male respondents shared that smoking cigarettes helps them to feel more relaxed and calm. A few participants prefer to get fresh air and see the cars go by outside their dorm to unwind. Participants do many activities with the nursing home staff, including festival celebrations, field trips, and university student and NGO visits. A few who were fit and healthy were allowed to go for an outing once every week, where they could go to nearby shops or shopping malls and buy outside food. Most participants said that having professional support to help and aid them helped them cope with their daily challenges. One participant said they got a monthly allowance from the government, which can be used to buy tasty food and other things (A001). A few participants noted that having medical personnel check on them and prescribe the medications made them less worried about their illnesses. There are visiting physiotherapists as well to aid participants who are bed-bound. Transport services are available to send them to nearby health facilities whenever they need. One participant said that the nursing home staff were friendly to them and helpful.

*“The staff here are okay too. Wherever I go, all the staff are nice… they speak politely with me and help a lot.”* (A009)

## Discussion

The findings of this study highlighted a multifaceted experience of loneliness shaped by personal loss and health-related, socio-economic, socio-cultural, and environmental factors. It also revealed the profound emotional and physical impacts of loneliness, along with the various coping strategies employed by the residents. The loss of loved ones was one of the most prominent factors contributing to loneliness. Participants mourned the death of parents, spouses, and children, often describing a lingering sense of emotional emptiness. This finding resonates with a previous study, which emphasized bereavement as a major precipitant of loneliness in older adults. [28]. Apart from that, economic vulnerability heightens loneliness, especially when it affects one’s ability to maintain familial roles or independence, which was reflected in the experiences of several participants who struggled after the loss of financial stability or employment [29].

Health-related issues, especially chronic diseases and physical limitations, were also frequently linked to loneliness. The inability to perform activities of daily living or pursue personal interests led to feelings of dependence and helplessness. This aligns with previous findings that reported that functional decline significantly reduces social participation, exacerbating isolation in older adults [30]. Socio-cultural and environmental challenges were also strongly implicated in the experience of loneliness. Several participants expressed that their family members had other commitments and were no longer actively involved.

Others reported a loss of connection with friends and the outside world. These findings align with earlier Malaysian studies, which reported that breakdowns in intergenerational relationships intensify feelings of loneliness among older adults [31]. In addition, the perception of living in ‘confinement’ or being ‘restricted’ within the nursing home was frequently voiced. Such confinement reflects structural limitations that can exacerbate loneliness when autonomy and community integration are hindered [32].

The impacts of loneliness were observed across both physical and emotional domains. Participants reported fatigue, poor appetite, sleep disturbances, and somatic complaints. Emotionally, they expressed sadness, anxiety, hopelessness, and a profound sense of loss. These findings echo previous work, which concluded that loneliness has comparable health risks to smoking and obesity, increasing mortality and morbidity [10]. The mental burden of feeling abandoned, missing their past lives, and anticipating a bleak future also reflects what a previous study described as the ‘psychological erosion’ that accompanies chronic loneliness in aging populations [33].

Participants employed a variety of strategies to cope with loneliness. Spiritual coping was particularly salient. Many turned to prayer, religious classes, and inner reflection, drawing strength and peace from their faith. This form of transcendental coping has been similarly reported from a previous study among older Muslims in Kuala Lumpur, Malaysia, where spirituality offers comfort and a framework for meaning-making in later life [34]. Strengthening social ties, whether through contact with family, bonding with fellow residents, or interactions with staff and visitors, emerged as a vital source of emotional support.

When effectively structured, institutional support can mitigate loneliness, a finding also supported by a previous study that advocated for relationship-centered care in long-term settings to address social isolation [35]. Distraction through hobbies, media, and structured activities was another major coping mechanism. Mobile devices and social media use to stay connected with the outside world were notable, suggesting the potential benefit of digital literacy programs in nursing homes. Some participants engaged in light physical activity, community chores, and outing activities, as shown in other studies, to enhance a sense of purpose and reduce depressive symptoms [36].

This study has a few limitations. Firstly, it was conducted in a single nursing home in the Northern region of Malaysia, which may limit the generalizability of the findings to other institutional settings with different organizational structures or resident demographics. However, qualitative studies are not about generalization. Secondly, the participants may have had recall or response biases, especially when discussing sensitive or emotional experiences. Lastly, as this was a cross-sectional exploration, the study could not evaluate changes in experiences of loneliness over time or concerning specific life events.

Future strategies should focus on enhancing family engagement through flexible visitation policies, the use of communication technologies, and the inclusion of families in nursing home activities and programs. Creating a socially stimulating environment with structured group activities and opportunities for peer interaction can foster a sense of belonging. Residents’ autonomy should be respected by allowing them to participate in daily decision-making and maintain personal routines, which may reduce feelings of confinement. Staff training on identifying and responding to emotional distress is essential to provide empathetic and psychosocial support. At the policy level, loneliness should be recognized as a public health concern, with appropriate funding allocated to initiatives that promote social inclusion and well-being within the institutionalized nursing homes.

## Conclusion

This study highlights that loneliness among institutionalized older adults is a multidimensional experience shaped by personal loss, declining health, socio-economic constraints, and institutional living. The emotional toll of loneliness is profound, with both physical and psychological consequences evident. However, the resilience shown by participants through spiritual strength, social connectedness, and meaningful activities offers valuable insight into potential interventions. These findings underscore the importance of holistic and culturally sensitive approaches in geriatric care by integrating emotional, spiritual, and social support within institutional settings.

## Abbreviations

USM: Universiti Sains Malaysia
SPSS: Statistical Package for the Social Sciences
NVIVO: brand name; software used for qualitative data analysis
CAQDA: Computer-Aided Qualitative Data Analysis
NGO: Non-Governmental Organization
SD: Standard Deviation
JEPeM: Jawatankuasa Etika Penyelidikan Manusia
CDC: Centers for Disease Control and Prevention
